# Improved Recovery of Exfoliated Colonocytes from Feces Using Newly Developed Immunomagnetic Beads

**DOI:** 10.1155/2008/605273

**Published:** 2008-12-22

**Authors:** Yoshikatsu Koga, Masahiro Yasunaga, Satoshi Katayose, Yoshihiro Moriya, Takayuki Akasu, Shin Fujita, Seiichiro Yamamoto, Hideo Baba, Yasuhiro Matsumura

**Affiliations:** ^1^Investigative Treatment Division, Research Center for Innovative Oncology, National Cancer Center Hospital East, 6-5-1 Kashiwanoha, Kashiwa 277-8577, Japan; ^2^Department of Gastroenterological Surgery, Graduate School of Medical Sciences, Kumamoto University, 1-1-1 Honjo, Kumamoto 860-8556, Japan; ^3^Tsukuba Research Laboratories, JSR Corporation, 25 Miyuki-gaoka, Tsukuba 305-0841, Japan; ^4^Department of Surgery, National Cancer Center Hospital, 5-1-1 Tsukiji, Chuo-ku, Tokyo 104-0045, Japan

## Abstract

We demonstrated the feasibility of a new methodology for isolating colonocytes from feces. To reduce costs and improve the recovery rate of colonocytes from feces, we attempted to develop new immunomagnetic beads. Several sizes of magnetic beads were prepared and tagged with a monoclonal antibody against EpCAM. We made several new monoclonal antibodies against EpCAM, and each monoclonal antibody was tagged to the magnetic beads. In the simulation, the most efficient recovery of HT-29 cells was obtained using the smallest size of beads. Also, beads tagged with a monoclonal antibody with a higher affinity against EpCAM had a higher recovery rate. Similar results were obtained when the smallest size of beads with the highest-affinity monoclonal antibody was applied to clinical samples. The newly developed immunomagnetic beads may be useful for isolating colorectal cancer cells from feces, enabling the cytological or molecular biological diagnosis of CRC.

## 1. INTRODUCTION

Colorectal cancer (CRC) is one of the most common malignancies worldwide. CRC is the third leading cause of cancer-related mortality and the second leading cause of cancer-related incidence. Nevertheless, the survival rate of patients with CRC is high if this cancer can be diagnosed and surgically resected at an early stage [1]. Thus, to reduce the mortality rate associated with CRC, the development of a screening test by which early-stage cancers can be detected is necessary.

The fecal occult blood test has been used widely as a screening test for CRC [2–4]. However, three recent large-scale studies have shown that the sensitivity of a fecal occult blood test was not very high when a total colonoscopy in all subjects was used as a reference standard [5–7]. Therefore, several new systems have been developed for diagnosing colorectal cancer based on the detection of mutated DNA in feces [8–19]. However, these methods are time consuming and are not sufficiently sensitive. The major reason for this inaccuracy is the fact that nucleic acids in feces are derived from an enormous number and a variety of bacteria and normal cells. Accordingly, the proportion of genes derived from cancer cells in feces is typically as low as 1%, at most [9]. This makes the clinical application of gene-detecting methods difficult.

Previously, we reported that cancer cells remain viable in the feces and can be isolated from naturally evacuated feces using an immunomagnetic bead methods that we developed [20, 21]. After extracting DNA from cells isolated from feces, CRC-related gene alterations were examined and a diagnostic sensitivity of 71% (82/116) and a specificity of 88% (73/83) were obtained [21].

These figures were relatively satisfactory, but the numbers of cells isolated from the feces were not very high and, in addition, commercially available immunomagnetic beads are relatively expensive. In this context, to improve the function of immunomagnetic beads for retrieving colonocytes from naturally evacuated feces and to reduce the cost performance of this method, we attempted to develop new immunomagnetic beads and to evaluate their usefulness.

## 2. MATERIALS AND METHODS

### 2.1. Immunomagnetic beads

**Figure 1:**
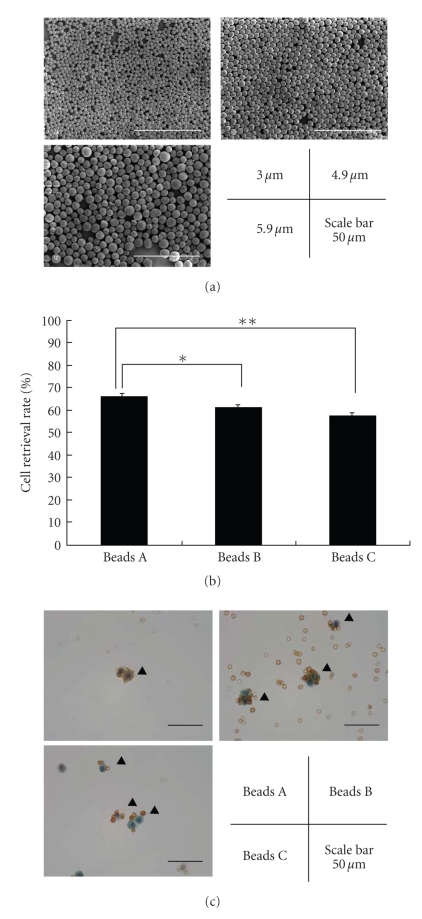
Simulation to evaluate the recovery rates of colonocytes using several sizes of beads. (a) Electron microscopy images show 3 different sizes of magnetic beads prepared by us. The sizes are 3.0, 4.9, and 5.9 *μ*m. Scale bar, 50 *μ*m. (b) Comparison of cell recovery rates. The cell recovery rates using beads A (smallest, 3.0 *μ*m), beads B (medium, 4.9 *μ*m), and beads C (largest, 5.9 *μ*m) are 65.9 ± 1.37 (%, mean ± SD), 61.1 ± 0.98, and 57.1 ± 0.75, respectively. The columns show the cell retrieval rates and the bars show the standard deviations. Significant differences are showed by an asterisk (∗, *P* = .0001) or a double asterisk (∗∗, *P* < .0001). (c) Papanicolaou staining of HT-29 cells captured using immunomagnetic beads. The arrowheads show the cell-bead complexes. The beads A have adhered to the HT-29 cells with the highest densely. Scale bar, 50 *μ*m.

First, we prepared three different sizes of magnetic beads (3.0, 4.9, and 5.9 *μ*m). Beads with each different size were tagged with commercially available antihuman EpCAM monoclonal antibody (mAb), VU-1D9 (AbD Serotec, Oxford, UK) (see Table 1). Briefly, magnetic beads for cell separation were prepared from polymer microspheres and supermagnetic iron oxide extracted from magnetic fluid. To make magnetic beads with different sizes, uniform polymer microspheres with diameters of 1.8, 3.3, or 3.9 *μ*m were coated with the iron oxide using mechanical shearing stress. The composite beads were then overcoated with a hydrophilic polymer layer through the polymerization of glycidyl methacrylate on the bead. After the hydrolysis of the poly glycidyl methacrylate layer, a tosyl group was introduced to the layer as an active group for antibody conjugation. The sizes of the obtained magnetic beads were determined to be 3.0, 4.9, and 5.9 *μ*m based on electron microscopic observation (see Figure 1(a)).

Immobilization of the antibody onto the beads was carried out using a 2-step reaction. First, an antimouse IgG mAb was coupled to the bead using the amino groups of the antibody. One hundred micrograms of the goat antimouse IgG (Fc; Millipore Corporation, Billerica, Ma, USA) were added to 1 mL of 1 wt% bead suspension in 0.1 M borate buffer, pH 9.5, and reacted for 24 hours at 37°C. After the elimination of uncoupled antibodies through repeated washing with TBS-T (25 mM Tris-buffered saline, pH 7.2, containing 0.01% Tween 20), anti-EpCAM mAb was immobilized on the bead as a second step. Twenty micrograms of commercially available anti-EpCAM mAb, VU-1D9, were added to 1 mL of 1 wt% bead in TBS-T and mixed for 1 hour at room temperature. After the reaction, uncaptured antibody was washed away with TBS-T and stored at 4°C until use.

Next, we developed new antihuman EpCAM mAbs in order to obtain an antibody with higher affinity against EpCAM. A recombinant human EpCAM/Fc chimera (R&D Systems, Minneapolis, Minn, USA) was used as an immunogen. The antigen (0.1 mg) was mixed with complete Freund's adjuvant (Difco, Detroit, Mich, USA) and injected intraperitoneally (IP) into BALB/c mice (Charles River Japan, Shizuoka, Japan). Subsequent injections were made using an RIBI adjuvant system, MPL + DM emulsion (Corixa, Seattle, Wash, USA) every three weeks for a total of 7 times. Three weeks later, the mice were given an intravenous (i.v.) booster injection of 0.1 mg of the antigen in phosphate buffered saline. Three days later, spleen cells from the immunized mice were fused with myeloma cells (P3X63Ag8.653) at a ratio of 7:1 in 50% polyethylene glycol 4000 (Sigma, St. Louis, Mo, USA) in RPMI 1640 at room temperature for 1 minute. After centrifugation, the cells were pelleted, washed, resuspended in RPMI 1640 containing 10% NCTC 109, 20% FCS, and 50 ng/L of mouse IL-6 (R&D Systems), and plated in flat-bottomed 96-well tissue culture palates (Costar Corning, Corning, NY, USA). Following overnight incubation in a humidified 5% CO_2_ atmosphere at 37°C, hypoxanthine-aminopterin-thymidine (HAT) medium was added to start HAT selection. Hybridoma clones were cultured for an additional 8 days, and then the culture media were assayed for specific antibody production using the ELISA method on Maxisorp microtiter plates (Nunc, Roskilde, Denmark) coated with the antigen. Briefly, an aliquot of undiluted hybridoma culture supernatant was added to the antigen coated wells. After 1 hour of incubation and a subsequent wash cycle, an appropriate dilution of peroxidase-conjugated rabbit IgG (Bethyl Laboratories, Montgomery, Ala, USA) to mouse IgG reagent was added. The enzyme-linked IgG binding was detected using an H_2_O_2_ and o-phenylenediamine substrate solution. The color intensity was measured automatically using a SpectraMax Plus microplate spectrophotometer (Molecular Devices, Sunnyvale, Calif, USA). The ELISA positive hybridoma cells were cloned by limiting dilution in 96-well culture plates and established as stable hybridoma cells.

The isotype of the mAb was determined using an MONO Ab-ID KIT (Zymed, San Francisco, Calif, USA) according to the manufacturer's instructions. Ascites fluids were obtained from pristine-primed CD1-Foxn1nu mice (Charles River Japan) injected with each mAb producing hybridoma clone. Immunoglobulin G was separately purified from each ascites fluid sample using protein G affinity chromatography (GE Healthcare Life Science, Piscataway, NJ, USA). The purified IgG fractions were used for further characterization and were evaluated for their reactivity. The immunoglobulin concentration was determined by measuring the absorbance at 280 nm using an extinction coefficient of 1.38 for mouse IgG.

The affinities to EpCAM of the newly developed antihuman EpCAM mAbs were analyzed using flow cytometry with a BD FACSCalibur (BD, Franklin Lakes, NJ, USA). HT-29 cells and UMUC-3 cells were used as an EpCAM positive control and negative control, respectively. The antihuman EpCAM mAbs and nonspecific mouse IgG1 were directly labeled using Zenon mouse IgG labeling kits (Molecular Probes, Eugene, Ore, USA) according to the manufacturer's instructions.

Finally, the new antihuman EpCAM mAbs were conjugated to the optimal size of magnetic beads chosen in the present study (see Table 1).

### 2.2. Simulation

A simulation was conducted to determine the optimal bead conditions for the recovery of HT-29 colorectal cancer cells using a previously established method [21]. Briefly, 2-gram fecal samples were homogenized in a Hanks-HEPES-FBS buffer (40 mL) consisting of Hanks solution, 10% fetal bovine serum (FBS), and 25 mM HEPES buffer (pH 7.35) at 200 times per minute for 1 minute using a Stomacher (Seward, Thetford, UK). A total of 1 × 10^5^ HT-29 cells were added to the homogenized solution and filtered through a nylon filter (pore size: 512 *μ*m). The HT-29 cells were retrieved using 80 *μ*L of several immunomagnetic beads, and the mixtures were incubated for 30 minutes under gentle rolling conditions at room temperature. The mixtures on the magnet were incubated on a shaking platform for 15 minutes at room temperature. Then, the supernatant was removed, and the retrieved cells were counted using a NucleoCounter (ChemoMetec A/S, Allerød, Denmark).

Finally, to determine the optimal immunomagnetic beads, HT-29 colorectal cancer cells were retrieved using these immunomagnetic beads, and then the cell-bead complexes were fixed and stained using papanicolaou stain.

### 2.3. Patients with colorectal cancer

From March 2007 to July 2008, 40 patients with histologically confirmed colorectal cancer were enrolled in the present study. Nineteen of the 40 patients were enrolled in a study to analyze the optimal bead size from March 2007 to September 2007. Then, 21 of the 40 patients were enrolled in a study to evaluate the quality of the antibody from October 2007 to July 2008. All the patients had undergone surgical resection of their primary cancer at the National Cancer Center Hospital, Tokyo, Japan. All patients were thoroughly informed of the content of the study, and provided their written consent to participate in the study. The study was approved by the Institutional Review Board of the National Cancer Center, Japan.

### 2.4. Clinical evaluation

Before surgical resection, naturally evacuated fecal samples were obtained from patients with colorectal cancer. Each fecal sample was divided into 2-gram samples for use in the evaluation of several immunomagnetic beads with different sizes and different affinities of the EpCAM antibody. The fecal samples were prepared as described in the simulation section. Colonocytes isolated from the feces were stored at −80°C until genomic DNA extraction.

Genomic DNA was extracted from colonocytes isolated from feces using an Allprep mini kit (QIAGEN, Valencia, Calif, USA) according to the manufacturer's instructions.

For the genomic DNA analysis, we targeted a consensus sequence of human Alu repeats. The sequences for the Alu primers and probe used in this study were as follows: forward primer, 5′-TAGTAGAGACGGGGTTTCACCTTG-3′; reverse primer, 5′-AGCTTGCAGTGAGCCGAGAT-3′; probe, 5′-GAGAATGGCGTGAA-3′. The reporter dye at the 5′-end of the probe was FAM, and the quencher dye at the 3′-end was MGB.

The reaction mixture for the genomic DNA analysis consisted of 4 *μ*L of a template DNA, 10 *μ*L of TaqMan Fast Universal PCR Master Mix (Applied Biosystems, Foster, Calif, USA), 500 nM of forward and reverse primers, and 250 nM of probe in a total reaction volume of 20 *μ*L. Real-time PCR amplification was performed using precycling heat activation at 95°C for 20 seconds, followed by 25 cycles of denaturation at 95°C for 3 seconds, and annealing/extension at 62°C for 30 seconds in an Applied Biosystems 7500 Fast Real-Time PCR System (Applied Biosystems). The absolute quantification of genomic DNA in each sample was determined using a standard curve with serial dilutions (10 ng to 100 fg) of TaqMan control genomic DNA (Applied Biosystems). A negative control (without template) was run in each reaction plate.

### 2.5. Statistical analysis

The cell retrieval rate for each group was analyzed using a Tukey-Kramer multiple comparisons test. Statistical differences in the cell retrieval abilities of the new immunomagnetic beads were determined using a two-sided Mann-Whitney U test. Statistical analyses were performed using StatView Ver. 5 for Windows (Abacus Concepts, Berkeley, Calif, USA). Values of *P* < .05 were considered statistically significant.

## 3. RESULTS

### 3.1. Recovery rates of colonocytes using several sizes of magnetic beads in the simulation study

We succeeded in preparing three different sizes of magnetic beads. The sizes of beads A, beads B, and beads C were 3.0, 4.9, and 5.9 *μ*m, respectively (see Table 1 and Figure 1(a)). To determine the best size of magnetic beads for cell recovery, various sizes of magnetic beads were tagged with a commercially available mAb, VU-1D9 (see Table 1). In the simulation, the cell recovery rates using beads A, B, and C were 65.9 ± 1.37 (%, mean ± SD), 61.1 ± 0.98, and 57.1 ± 0.75, respectively. The recovery rate using beads A (the smallest size) was significantly higher than those using beads B (*P* = .0001) and beads C (*P* < .0001) (see Figure 1(b)). Microscopic observation also showed that the HT-29 cells were captured using the newly developed immunomagnetic beads, and that more HT-29 cells were bound to beads A than to the larger beads B and beads C (see Figure 1(c)).

### 3.2. Newly developed anti-EpCAM monoclonal antibodies

**Figure 2:**
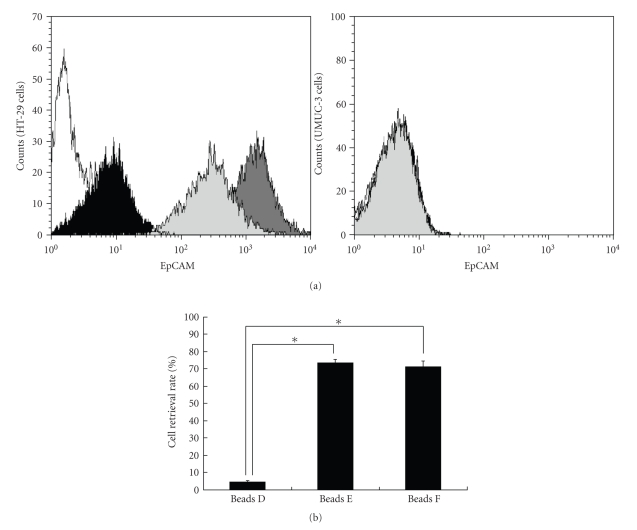
A simulation experiment for the recovery rates of colonocytes depending on the affinity of mAbs. (a) The affinities of newly developed antihuman EpCAM mAbs are analyzed using flow cytometry. HT-29 cells, which are positive for EpCAM antigen, and UMUC-3 cells, which are negative for EpCAM antigen, are used. The white peak, black peak, dark-gray peak, and light-gray peak show nonspecific mouse IgG1 mAb, clone 1-2, clone B8-4, and clone B8-7, respectively. (b) Simulation examining the recovery of the colonocytes. The cell recovery rates using beads D (tagged with a lower affinity mAb, Clone 1-2), beads E (tagged with a higher affinity mAb, Clone B8-4), and beads F (tagged with a higher affinity mAb, Clone B8-7) are 4.5 ± 0.98 (%, mean ± SD), 73.5 ± 1.96, and 71.2 ± 3.39, respectively. The columns show the cell retrieval rates and the bars show the standard deviations. Significant differences are showed by an asterisk (∗, *P* < .0001).

We developed three new anti-EpCAM mAbs, named clone 1-2, clone B8-4, and clone B8-7. Flow cytometry analysis using HT-29 cells (positive for the EpCAM antigen) showed that the affinity of clone 1-2 was 3 times higher than that of nonspecific mouse IgG1, while the affinities of clones B8-4 and B8-7 were 500 times higher than that of nonspecific mouse IgG1 (see Figure 2(a)). The affinity intensities of clones 1-2, B8-4, and B8-7 to UMUC-3 cells (negative for EpCAM antigen) were almost identical to that of nonspecific mouse IgG1 to UMUC-3 cells. These results show that clone 1-2 was a low-affinity mAb and that clones B8-4 and B8-7 were high-affinity mAb (see Figure 2(a)). Each antibody was then conjugated to the optimal size (3.0 *μ*m) of magnetic beads (see Table 1).

### 3.3. Recovery rate of colonocytes depending on the affinity of monoclonal antibodies against EpCAM in the simulation study

In the simulation, the cell recovery rates using beads D, E, and F were 4.5 ± 0.98 (%, mean ± SD), 73.5 ± 1.96, and 71.2 ± 3.39, respectively. The recovery rate using beads D was significantly lower than those using beads E (*P* < .0001) and beads F (*P* < .0001) (see Figure 2(b)).

### 3.4. Cell retrieval ability of newly developed immunomagnetic beads in clinical samples

**Figure 3:**
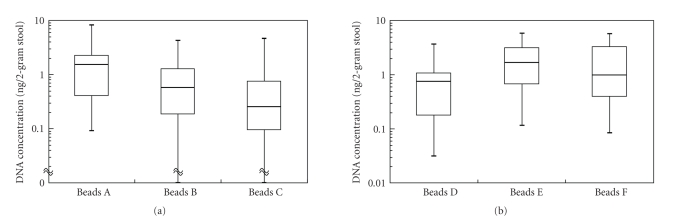
Cell retrieval ability of newly developed immunomagnetic beads in clinical samples. (a) Cell retrieval ability using several sized immunomagnetic beads in clinical samples. The median amounts of DNA from 2-gram stool using beads A (smallest, 3.0 *μ*m), beads B (medium, 4.9 *μ*m), and beads C (largest, 5.9 *μ*m) are 1.50 ng (range; 0.09–8.12), 0.58 ng (range; 0–4.23), and 0.25 ng (range; 0–4.62), respectively. The horizontal bar is a median amount of extracted DNA from isolated colonocytes from 2-gram stool; the upper vertical bar is a maximal amount of extracted DNA; the lower vertical bar is a minimal amount of extracted DNA; and the column contained 50% of these populations. (b) Cell retrieval ability using immunomagnetic beads tagged with several affinities mAbs in clinical samples. The median amounts of DNA from 2-gram stool using beads D (tagged with a lower affinity mAb, Clone 1-2), beads E (tagged with a higher affinity mAb, Clone B8-4), and beads F (tagged with a higher affinity mAb, Clone B8-7) are 0.75 ng (range; 0.03–3.65), 1.66 ng (range; 0.12–5.74), and 0.99 ng (range; 0.08–5.67), respectively. The horizontal bar is a median amount of extracted DNA from isolated colonocytes from 2-gram stool; the upper vertical bar is a maximal amount of extracted DNA; the lower vertical bar is a minimal amount of extracted DNA; and the column contained 50% of these populations.

In the clinical study examining cell retrieval ability according to bead size, the median amount of DNA from 2-gram stool using beads A, B, and C was 1.50 ng (range 0.09–8.12), 0.58 ng (range 0–4.23), and 0.25 ng (range 0–4.62), respectively (see Figure 3(a)). The amount of extracted DNA using beads A (the smallest beads) was not significantly different from that using beads B (*P* = .09). However, the amount of extracted DNA using beads A was significantly higher than that using beads C (*P* = .02). Meanwhile, in the clinical study examining cell retrieval ability according to antibody affinity, the median amount of DNA from 2-gram stool using beads D, E, and F was 0.75 ng (range 0.03–3.65), 1.66 ng (range 0.12–5.74), and 0.99 ng (range 0.08–5.67), respectively (see Figure 3(b)). The amount of extracted DNA using beads D was significantly less compared with that using beads E (*P* = .01).

These results clarified that the smaller immunomagnetic beads and the beads that were conjugated with higher-affinity Abs were more efficient at retrieving colonocytes from feces.

## 4. DISCUSSION

CRC develops from the colorectal mucosa and therefore is in continuous contact with feces from an early stage. Previously, we proposed that colorectal cancer cells exfoliated into the feces might survive for a considerable period in the feces, since feces do not inhibit cancer expansion towards the colorectal lumen [20–22]. Meanwhile, we predicted that normal colorectal mucous cells in contact with the feces would be in an apoptotic stage and would probably be exfoliated into the feces. To date, therefore, we have tried to develop a method by which exfoliated colorectal cancer cells can be isolated from naturally evacuated feces and then utilized in cytological or molecular biological diagnosis [21]. In the present study, we developed new immunomagnetic beads and evaluated their efficiencies in terms of the recovery of exfoliated colorectal cancer cells in feces because we had recognized an urgent need to improve the accuracy of this method of isolating colonocytes from feces and to reduce the cost performance.

Intuitively, larger magnetic beads seem more likely to be attracted to magnetic body, compared with smaller magnetic beads, since their larger size corresponds to a greater number of iron particles inside the bead. Therefore, we initially thought that more cell-bead complexes using the larger magnetic beads would adhere to the magnetic body, enabling more cells to be collected. However, in a study to determine the optimal bead size, the smaller beads adhered to the HT-29 cells more densely than the larger beads (see Figure 1(c)). Thus, the total number of cell-bead complexes attracted to the magnetic body was significantly higher when the smaller beads were used. The cell retrieval rate using the smaller beads was significantly higher than those using the larger beads (see Figure 1(b)). The results of an experiment using clinical samples were similar to those obtained in the simulation (see Figure 3(a)). Consequently, the smallest beads appeared to collect colonocytes from feces more efficiently than the larger beads.

In our preliminary experiments using a flow cytometry, it was found that EpCAM antigen was expressed strongly in human colorectal cancer cell lines, DLD-1, HCT116, HCT-15, HT-29, LoVo, and SW480. We also analyzed the positivity of cancer tissue and normal mucosal tissue using immunohistochemistry. Both the cancer tissues and the normal mucosal tissues were strongly positive (data not shown). We then made several mAbs against human EpCAM and consequently obtained mAbs with either a high affinity or a low affinity to the EpCAM antigen (see Figure 2(a)). Each antibody was conjugated to the optimum (the smallest) beads, and these immunomagnetic beads were compared with each other. In a simulation using feces seeded with 1 × 10^5^ HT-29 cells, the cell retrieval rate using immunomagnetic beads tagged with a high-affinity antibody was superior to that of the beads tagged with a low-affinity antibody (see Figure 2(b)). This result indicated that the higher-affinity antibody tagged to the magnetic beads was useful for retrieving the cells present in fecal samples. In addition, the results of a clinical study were similar to the results of the simulation (see Figure 3(b)).

Previously, we demonstrated the feasibility of a new methodology for isolating colonocytes from naturally evacuated feces, followed by cytological or molecular biological analysis of the colonocytes to detect colorectal cancer [21]. An improvement in the immunomagnetic beads was the most important issue for detecting colorectal cancer using our diagnostic method. Dynabeads Epithelial Enrich, immunomagnetic beads for isolating free circulating cancer cells from serum, was used in our original method. However, immunomagnetic beads developed for the collection of colorectal cancer cells from stool solutions, which contain large amounts of residual substances, were not available. Therefore, we decided to develop immunomagnetic beads designed especially for the isolation of colorectal cancer cells from feces.

## 5. CONCLUSIONS

We succeeded in developing suitable immunomagnetic beads for the isolation of colonocytes from feces. The new immunomagnetic beads, which were 3.0 *μ*m in size and were conjugated with a new monoclonal antibody possessing a higher affinity to EpCAM, could effectively isolate colonocytes from feces. This result is promising for the future of CRC diagnosis.

## Figures and Tables

**Table 1:** New immunomagnetic beads.

Beads	Size (*μ*m)	Antihuman EpCAM antibodies
A	3.0	VU-1D9
B	4.9	VU-1D9
C	5.9	VU-1D9
D	3.0	Clone1-2
E	3.0	Clone B8-4
F	3.0	Clone B8-7

VU-1D9, commercially available EpCAM antibody; clone1-2, clone B8-4, and clone B8-7 were developed by us in the present study.

## References

[1] Bosman FT (1995). Prognostic value of pathological characteristics of colorectal cancer. *European Journal of Cancer*.

[2] Towler B, Irwig L, Glasziou P, Kewenter J, Weller D, Silagy C (1998). A systematic review of the effects of screening for colorectal cancer using the faecal occult blood test, Hemoccult. *British Medical Journal*.

[3] Winawer S, Fletcher R, Rex D (2003). Colorectal cancer screening and surveillance: clinical guidelines and rationale—update based on new evidence. *Gastroenterology*.

[4] Mandel JS, Church TR, Bond JH (2000). The effect of fecal occult-blood screening on the incidence of colorectal cancer. *The New England Journal of Medicine*.

[5] Lieberman DA, Weiss DG (2001). One-time screening for colorectal cancer with combined fecal occult-blood testing and examination of the distal colon. *The New England Journal of Medicine*.

[6] Sung JJY, Chan FKL, Leung WK (2003). Screening for colorectal cancer in Chinese: comparison of fecal occult blood test, flexible sigmoidoscopy, and colonoscopy. *Gastroenterology*.

[7] Imperiale TF, Ransohoff DF, Itzkowitz SH, Turnbull BA, Ross ME (2004). Fecal DNA versus fecal occult blood for colorectal-cancer screening in an average-risk population. *The New England Journal of Medicine*.

[8] Sidransky D, Tokino T, Hamilton SR (1992). Identification of ras oncogene mutations in the stool of patients with curable colorectal tumors. *Science*.

[9] Hasegawa Y, Takeda S, Ichii S (1995). Detection of K-ras mutations in DNAs isolated from feces of patients with colorectal tumors by mutant-allele-specific amplification (MASA). *Oncogene*.

[10] Smith-Ravin J, England J, Talbot IC, Bodmer W (1995). Detection of c-Ki-ras mutations in faecal samples from sporadic colorectal cancer patients. *Gut*.

[11] Eguchi S, Kohara N, Komuta K, Kanematsu T (1996). Mutations of the p53 gene in the stool of patients with resectable colorectal cancer. *Cancer*.

[12] Nollau P, Moser C, Weinland G, Wagener C (1996). Detection of K-ras mutations in stools of patients with colorectal cancer by mutant-enriched PCR. *International Journal of Cancer*.

[13] Ratto C, Flamini G, Sofo L (1996). Detection of oncogene mutation from neoplastic colonic cells exfoliated in feces. *Diseases of the Colon and Rectum*.

[14] Deuter R, Müller O (1998). Detection of APC mutations in stool DNA of patients with colorectal cancer by HD-PCR. *Human Mutation*.

[15] Ahlquist DA, Skoletsky JE, Boynton KA (2000). Colorectal cancer screening by detection of altered human DNA in stool: feasibility of a multitarget assay panel. *Gastroenterology*.

[16] Dong SM, Traverso G, Johnson C (2001). Detecting colorectal cancer in stool with the use of multiple genetic targets. *Journal of the National Cancer Institute*.

[17] Rengucci C, Maiolo P, Saragoni L, Zoli W, Amadori D, Calistri D (2001). Multiple detection of genetic alterations in tumors and stool. *Clinical Cancer Research*.

[18] Traverso G, Shuber A, Olsson L (2002). Detection of proximal colorectal cancers through analysis of faecal DNA. *The Lancet*.

[19] Traverso G, Shuber A, Levin B (2002). Detection of APC mutations in fecal DNA from patients with colorectal tumors. *The New England Journal of Medicine*.

[20] Yamao T, Matsumura Y, Shimada Y (1998). Abnormal expression of CD44 variants in the exfoliated cells in the feces of patients with colorectal cancer. *Gastroenterology*.

[21] Matsushita H, Matsumura Y, Moriya Y (2005). A new method for isolating colonocytes from naturally evacuated feces and its clinical application to colorectal cancer diagnosis. *Gastroenterology*.

[22] Onouchi S, Matsushita H, Nomura S, Minowa T, Matsumura Y (2007). PCR-based assessment of the recovery rate of exfoliated colonocytes or cancer cells from fecal samples depends on the storage conditions after defecation. *Journal of Gastrointestinal and Liver Diseases*.

